# Changing the phospholipid composition of lipid droplets alters localization of select lipid droplet proteins

**DOI:** 10.17912/micropub.biology.000960

**Published:** 2023-11-03

**Authors:** Timothy J. Renier, Olivia R. Paetz, Matthew C. Paal, Alex B. Long, Margaret R. Brown, Sunny H. Vuong, Sathish Kumar Perumal, Kusum K. Kharbanda, Laura L. Listenberger

**Affiliations:** 1 Departments of Biology and Chemistry, St. Olaf College, Northfield, Minnesota, United States; 2 Department of Internal Medicine, University of Nebraska Medical Center, Omaha, Nebraska, United States; 3 Research Service, Veterans Affairs Nebraska-Western Iowa Health Care System, Omaha, Nebraska, United States; 4 Department of Biochemistry & Molecular Biology, University of Nebraska Medical Center, Omaha, Nebraska, United States

## Abstract

Our experiments aim to determine if decreasing the amount of phosphatidylcholine (PC) relative to phosphatidylethanolamine (PE) at the lipid droplet surface changes the localization of specific lipid droplet proteins. We manipulate lipid droplet phospholipids in both a cultured mouse hepatocyte (AML12) cell line and on synthetic lipid droplets. Decreasing the PC:PE ratio increases perilipin 2, decreases DGAT2, and does not change rab18 or lanosterol synthase levels on lipid droplets. These differences may be explained by the distinct structural motifs that mediate the protein-lipid droplet interactions.

**Figure 1. Manipulation of lipid droplet phospholipid composition and localization of lipid droplet proteins:**
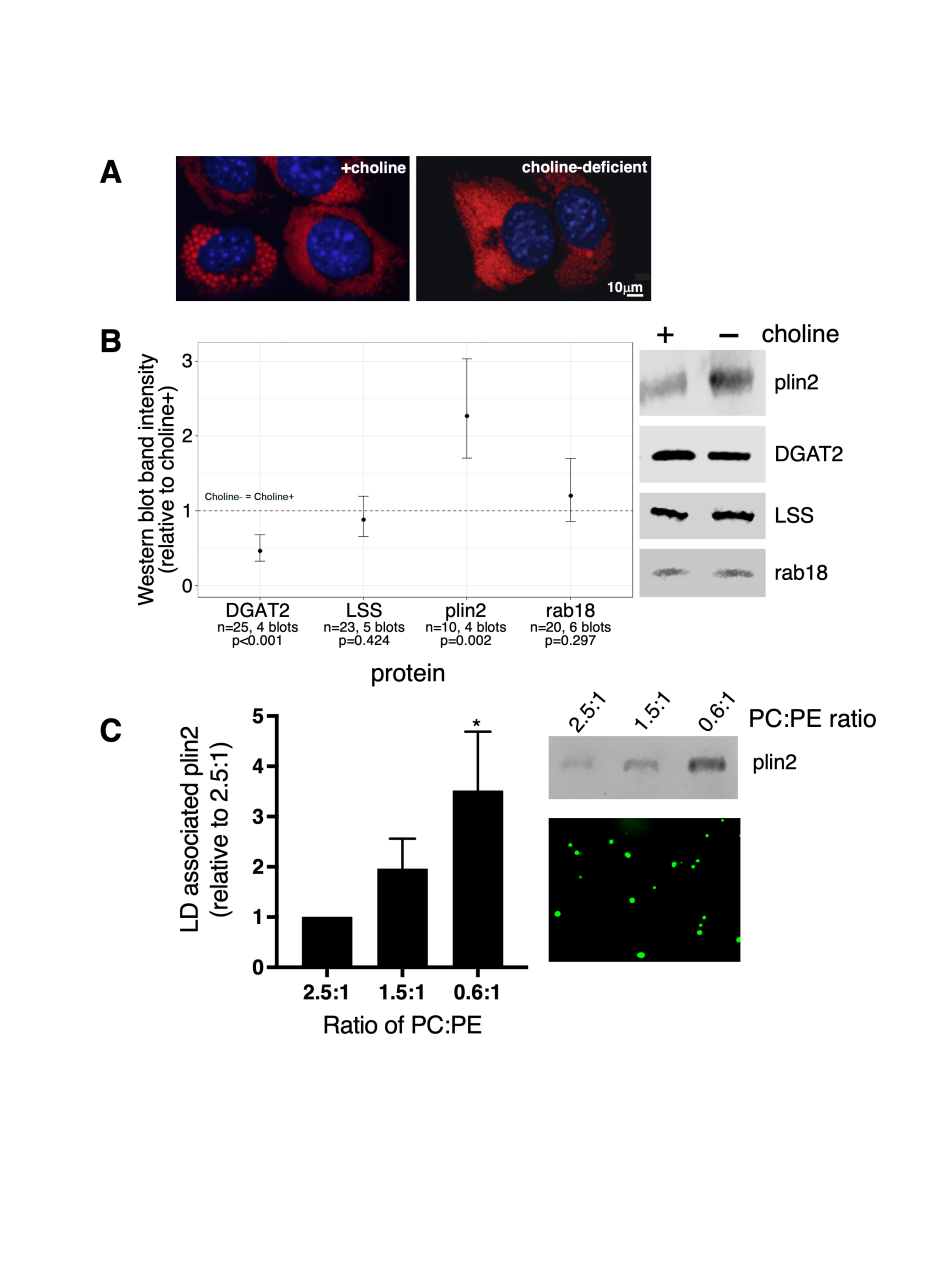
(A) AML12 cells were supplemented with 500 μM oleate for 24 hours in media ± choline. We have previously shown that choline deficiency decreases the relative amount of PC to PE on lipid droplets (Listenberger et al., 2018). BODIPY 493/503 stained lipid droplets are shown pseudo-colored red. Hoechst stained nuclei are shown in blue. We observed abundant lipid accumulation under both growth conditions. Images are of representative cells showing the heterogeneity in lipid droplet size that is common in cultured cells. (B) Lipid droplets were isolated from AML12 cells supplemented with oleate and grown in media ± choline. Proteins of interest were detected by SDS-PAGE and western blotting. Each blot contained at least one lane of choline-deficient and -sufficient lipid droplets, with equal total protein concentration for all lanes within a blot. The estimates shown are exponentiated betas from mixed-effects linear regression models of log-transformed western blot band pixel intensities by choline condition, accounting for each western blot with a random intercept. Estimates are interpreted as the multiplicative difference in intensity for choline-deficient relative to choline-sufficient lanes, accounting for the random effect. Error bars are 95% confidence intervals for regression estimates and p-values correspond to the main effect term for choline condition in the regression model of each protein. Representative western blots for each lipid droplet protein are shown. plin2, perilipin 2; DGAT2, acyl-CoA:diacylglycerol acyltransferase 2; LSS, lanosterol synthase. (C) To evaluate perilipin 2 association with model lipid droplets of defined phospholipid composition, we measured perilipin 2 abundance in floating lipid droplet (LD) fractions following an *in vitro* binding reaction between synthetic lipid droplets and partially purified, soluble perilipin 2 from a stable overexpressing HEK293 cell line. Data are expressed relative to the amount of perilipin 2 detected in floating fractions when lipid droplets were synthesized with a 2.5:1 PC:PE ratio. The graph shows means ± standard error. Of eight total experiments, six compared 0.6:1 to 2.5:1, and seven compared 1.5:1 to 2.5:1. (*, p=0.031, Wilcoxon signed-rank test for 0.6:1 relative to 2.5:1). A representative western blot of perilipin 2 (plin2) in equivalent amounts of floating lipid droplet fractions and an image of the synthetic lipid droplets stained with BODIPY 493/503 (green) are shown.

## Description

Excess lipid is stored in intracellular organelles known as lipid droplets. Each lipid droplet consists of a neutral lipid core surrounded by a phospholipid monolayer decorated with proteins. Class I or ERTOLD proteins localize to the lipid droplet from the ER membrane as the lipid droplet forms and buds from the ER (Dhiman et al., 2020; Olarte et al., 2021) . These proteins contain monotopic hydrophobic domains that mediate interaction with both the ER and lipid droplet surface. Class II or CYTOLD proteins associate with the lipid droplet following translation in the cytosol (Dhiman et al., 2020; Olarte et al., 2021) . These soluble proteins bind the lipid droplet surface through amphipathic alpha helices, lipid-anchors, or association with other embedded lipid droplet proteins. Our experiments explore how changes to the phospholipid composition of lipid droplets impact localization of different classes of lipid droplet proteins.

Severe over-accumulation of lipid droplets in the liver is the hallmark of alcohol-associated fatty liver disease (AFLD). We examined the composition of lipid droplets from the livers of rats fed an ethanol-enriched diet and reported a decrease in the ratio of phosphatidylcholine (PC) to phosphatidylethanolamine (PE), two phospholipids commonly associated with the lipid droplet surface (Listenberger et al., 2018) . This difference in phospholipid composition likely results from decreased synthesis of PC from PE via an enzymatic pathway requiring phosphatidylethanolamine *N-* methyltransferase (PEMT) (Barak et al., 2003; Kharbanda et al., 2007) . We hypothesize that changes to the phospholipid surface of lipid droplets may subsequently alter protein binding.

Our laboratory developed a cell culture system to explore changes to the phospholipid composition of lipid droplets in cultured cells. We have previously shown that culturing the mouse hepatocyte AML12 cell line in choline-deficient media decreases the PC:PE ratio on lipid droplets by 50% (Listenberger et al., 2018) . Lipid droplets isolated from these choline-deficient cells had increased levels of perilipin 2 and 3, two well-studied CYTOLD proteins that associate with lipid droplets through amphipathic alpha helices. Despite an increase in perilipin 2 and 3 on lipid droplets, the total amount of lipid droplet protein did not differ between choline ± conditions (Listenberger et al., 2018) .

Here, we expand this work to explore additional ERTOLD and CYTOLD lipid droplet proteins. We intentionally chose proteins that interact with lipid droplets through distinct structural motifs. Rab18 is a soluble protein that associates with lipid droplets via a mechanism that may involve a lipid anchor or binding to other lipid droplet proteins (Li et al., 2017; Deng et al., 2021) . Whether and how rab18 impacts lipid droplet function is unclear and may be cell-type specific, although there is some evidence suggesting a role in facilitating lipid droplet-ER contacts (Jayson et al., 2018; Hugenroth et al., 2020) . Lanosterol synthase is a monotopic membrane protein that catalyzes an important step in cholesterol biosynthesis. The crystal structure of this protein has identified a hydrophobic domain that is thought to mediate association with the cytosolic face of the ER (Thoma et al., 2004) . Lanosterol synthase has also been identified as a lipid droplet protein (Fujimoto et al., 2003; Liu et al., 2003; Brasaemle et al., 2004) . While the lipid droplet binding domain for lanosterol synthase has not been verified, it likely includes the same hydrophobic domain that facilitates association with the ER. DGAT2 catalyzes the final step in triacylglycerol synthesis. The structure of this protein includes two transmembrane domains that anchor the protein in the ER, and a C-terminal domain that mediates binding to lipid droplets (McFie et al., 2018) . The C-terminal binding domain is predicted to fold into an amphipathic alpha helix (McFie et al., 2018) .

To determine if lipid droplet phospholipid composition impacts localization of these proteins, we induced lipid droplet accumulation in AML12 cells by supplementation with 500 µM oleate under either choline-sufficient or -deficient conditions. Oleate supplementation led to massive lipid droplet accumulation under both growth conditions ( Fig. 1A ). While there was heterogeneity in lipid droplet size and number within each population, we did not detect differences in the overall pattern of lipid droplet accumulation between cell treatments ( Fig. 1A ). Lipid droplets were collected from cultured cells following flotation on a sucrose gradient and proteins of interest were detected in equivalent amounts of total lipid droplet protein ( Fig. 1B ). We repeated and confirmed our published results (Listenberger et al., 2018) showing an increase in perilipin 2 association with lipid droplets with reduced PC:PE ratios, that is, from choline-deficient growth conditions ( Fig. 1C ). Additionally, similar levels of two proteins, rab18 and lanosterol synthase, were found on lipid droplets under both choline-sufficient and -deficient conditions. On the other hand, we found 53% less of a third protein, DGAT2, in lipid droplet fractions from the choline-deficient cells.

We next aimed to further explore the increase in perilipin 2 association with lipid droplets from choline-deficient cells, using an *in vitro* binding assay with partially purified proteins from over-expressing cell lines and model lipid droplets with specific ratios of PC:PE. We previously published data from an earlier version of this assay showing increased perilipin 2 binding to large unilamellar vesicles as relative levels of PC decreased (Listenberger et al., 2018) . Here, we create an assay that better mimics the structure of the lipid droplet, with synthetic lipid droplets constructed with phospholipid monolayers surrounding triglyceride cores rather than unilamellar vesicles with a phospholipid bilayer. Our new experiments continue to support increasing perilipin 2 association as the amount of PC relative to PE decreases ( Fig. 1C ). PC:PE ratios on hepatic lipid droplets can vary widely (Arumugan et al., 2023). Preliminary biochemical analysis on size-based sub-populations of lipid droplets isolated from the livers of rats fed the ethanol Lieber DeCarli diet for five weeks revealed that the largest lipid droplets have the lowest PC:PE ratio (0.6:1), which increased inversely with lipid droplet size. In our *in vitro* binding experiments, synthetic lipid droplets with elevated ratios of PC to PE (3.5:1 and 4.5:1) showed little to no perilipin 2 association and were therefore not useful as comparisons in these experiments.

Our data contribute to a growing body of evidence describing the relationship between the phospholipid and protein composition of lipid droplets (Krahmer et al., 2011; Mirheydari et al., 2016) . Recent studies have demonstrated that exposed underlying hydrophobic lipid, or “packing defects”, in the lipid droplet phospholipid monolayer may serve as insertion points for amphipathic alpha helical motifs(Prevost et al., 2018). Because the head group of PE is smaller than PC, we propose that this change in the lipid droplet phospholipid composition increases the presence of packing defects and thus increases the available binding sites for some lipid droplet proteins. Importantly, this change in phospholipid composition and presumed effect on surface packing does not impact association of all lipid droplet proteins. Our results indicate that the amphipathic alpha helix-containing proteins perilipin 2 (and perilipin 3 as included in Listenberger et al., 2018) are significantly more likely to associate with lipid droplets with decreased PC:PE ratios. This same change in the surface phospholipid composition did not increase lipid droplet binding for proteins that are predicted to interact with lipid droplets through a lipid anchor (rab18), hydrophobic patch (lanosterol synthase), or those that may connect simultaneously with the ER and lipid droplet (DGAT2).

Our data suggest proteins that are translated in the cytosol and subsequently interact with lipid droplets through amphipathic alpha helices are more likely to interact with lipid droplets with decreased PC:PE levels. In contrast, proteins that bind to lipid droplets through other motifs (like lipid anchors) or that traffic to the lipid droplet following association with the ER fail to be recruited to lipid droplets with decreased PC:PE. Thus, it may be possible to predict how a specific lipid droplet protein will respond to changes in the phospholipid composition of lipid droplets if the mechanism of localization to lipid droplets is known.

Future experiments should explore whether the relationship between lipid droplet phospholipid composition and the association of specific proteins contributes to AFLD, or other disorders of altered lipid droplet accumulation. Because perilipin 2 is known to promote lipid droplet accumulation via inhibiting lipolysis (Brasaemle et al., 1997; Imamura et al., 2002; Magnusson et al., 2006; Listenberger et al., 2007) , it is possible that the change in lipid droplet phospholipid composition observed in ethanol-fed rats drives the development of hepatic steatosis through perilipin 2 recruitment. On the other hand, we see decreased levels of DGAT2, an enzyme involved in triglyceride synthesis. The functional consequences of changes to lipid droplet phospholipid and protein composition are likely to be complex.

**Abbreviations:** AFLD, alcohol-associated fatty liver disease; ATCC, American type culture collection; CYTOLD, cytosol to lipid droplet; DGAT2, acyl-CoA:diacylglycerol acyltransferase 2; DMEM, Dulbecco’s modified Eagle medium; ERTOLD, endoplasmic reticulum to lipid droplet; LSS, lanosterol synthase; PC, phosphatidylcholine; PE, phosphatidylethanolamine; PEMT, phosphatidylethanolamine *N-* methyltransferase; plin2, perilipin2.

## Methods

*Cell Culture*

AML12 cells (from ATCC) were cultured in DMEM (Gibco #11965) with 10% fetal bovine serum, 1% penicillin/streptomycin and 0.1% insulin-transferrin-selenium. For choline-deficiency, media was prepared with a custom formulation of GIBCO #11965 DMEM lacking choline chloride. Lipid droplet accumulation was induced by supplementing either choline-sufficient or -deficient cell culture media with 500 µM oleate complexed to bovine serum albumin as described (Listenberger et al., 2001) .

Because endogenous levels of DGAT2 in AML12 cells were too low to be reliably detected, we created DGAT2 overexpressing cells by transfecting AML12 cells with the pCMV3-mDGAT2-Flag plasmid (Sino Biological MG58866-CF) with Lipofectamine 2000 (Invitrogen 11668-030). All experiments examining DGAT2 were performed in a stable population of transfected cells selected with G418.

*Microscopy*

AML12 cells were seeded onto coverslips and, on the following day, were supplemented with 500 µM oleate in the presence or absence of choline. After 24 hours, the media was replaced with the same oleate supplemented ±choline media, additionally supplemented with 5µg/ml BODIPY 493/503 and 1µg/ml Hoechst dye. After one hour, the media was again replaced with fresh oleate supplemented ±choline media and coverslips were mounted on slides. Hoechst-stained nuclei (blue) and BODIPY 493/503-stained lipid droplets (pseudo-colored red) were detected with an Olympus Bx53 fluorescent microscope and images overlayed with cellSens Dimension software.

*Isolation of Lipid Droplets from Cultured Cells*

AML12 cells were supplemented with 500 µM oleate in the presence or absence of choline for 24-48 hours. Lipid droplets were isolated as previously described (Listenberger et al., 2018) . Briefly, cells lysed and lipids were collected as a floating lipid layer following density-gradient centrifugation. Total protein concentrations in collected fractions were determined using a Bradford Assay (Bradford, 1976) .

*SDS-PAGE and Immunoblotting*

Equivalent amounts of total lipid droplet associated proteins were resolved by SDS-PAGE with 10% polyacrylamide gels. Proteins were transferred to nitrocellulose membrane and detected with rabbit polyclonal antibodies to perilipin 2 (Novus Biologicals NB110-40877), rab18 (OWL, 58637), lanosterol synthase (Fitzgerald Industries, 7OR-2748), or DGAT2 (Novus Biologicals, NB600-345) and LiCor IR Dye 800CW goat anti-rabbit secondary antibodies (LiCor, 926-32211). Blots were imaged with a LiCor Odyssey imager and band intensity was determined by ImageJ.

*Synthetic lipid droplets preparation and visualization*

Model lipid droplets were prepared by mixing 70 µl triolein with 0.5 µmol phospholipids (2.5, 1.5, or 0.6:1 PC:PE) in chloroform. Mixtures were dried under nitrogen and incubated at 70ºC for 10 minutes. Dried lipids were resuspended in HEPES buffer (10 mM HEPES, 5 mM EDTA, pH 7.4), incubated at 70ºC for 10 minutes, and further hydrated on a shaking platform at 50ºC for 30 minutes. Samples were vortexed three times at maximum speed, and sonicated on ice for 1 min (amplitude 25). An aliquot of artificial lipid droplets was stained with 10 µg/ml BODIPY 493/503 and visualized using fluorescence microscopy.

*In Vitro Binding Assay*

Cytosol enriched for a soluble fraction of perilipin 2 was isolated from perilipin 2-overexpressing HEK293 cells as previously described (Listenberger et al., 2018) . This fraction of soluble perilipin 2 was distributed into tubes with synthetic lipid droplets and incubated in an orbital mixer for 1 hour at room temperature. Samples were adjusted to 25% sucrose, loaded into centrifuge tubes, overlaid with HEPES buffer and centrifuged at 280,000xg, 4ºC, for a minimum of 3 hours. Synthetic lipid droplets and associated proteins were collected from the top of each tube. In order to normalize the concentrations of collected lipid droplets, we used a Nanodrop 2000 to quantify turbidity. Samples were diluted with 15% sucrose in HEPES buffer when necessary to achieve overlapping spectra. Perilipin 2 was detected in samples of equivalent turbidity by SDS-PAGE and immunoblotting.

*Statistical Analysis*

Multiple replicates of the experiment were performed for each protein examined for Figure 1B, with multiple lanes for each choline condition on each western blot within each replicate . Protein band intensities from western blots were log-transformed due to deviation from normal distribution. To account for the dependence of multiple band intensity observations within the same western blot and retain statistical power, a mixed-effects linear regression model for each protein assessed log-transformed protein band intensities by choline condition with a random intercept for each replicate of the experiment. Any blot that did not yield sufficient pixel intensity to quantify both choline conditions was excluded from analysis. Statistical significance was defined as *p < 0.05* . All regression modeling was conducted with the R language for statistical computing (R Core Team, 2023).

A different statistical strategy was adopted for analysis and representation of the data in Figure 1C . Pixel intensities of bands on images of western blots (n=8) were determined with ImageJ and expressed relative to the intensity of the 2.5:1 sample band. Means and standard errors of these pixel intensities were calculated and the figure created with GraphPad Prism version 10.1.0 for macOS (GraphPad Software, Boston, Massachusetts USA, www.graphpad.com). We also used GraphPad Prism for the Wilcoxon signed-rank test to compare the intensities of the 1.5:1 band to 1.0 (the relative intensity of the 2.5:1 band) and, separately, to compare the intensities of the 0.6:1 samples to 1.0. Only the p-value for the comparison that included the 0.6:1 samples was significant (<0.05) and is reported.
